# Chlamydial Lung Infection Induces Transient IL-9 Production Which Is Redundant for Host Defense against Primary Infection

**DOI:** 10.1371/journal.pone.0115195

**Published:** 2015-02-03

**Authors:** Ying Peng, Xiaoling Gao, Jie Yang, Sudhanshu Shekhar, Shuhe Wang, Yijun Fan, Xi Yang

**Affiliations:** Departments of Immunology and Medical Microbiology, University of Manitoba, Winnipeg, Manitoba, Canada; University of California Merced, UNITED STATES

## Abstract

IL-9/Th9 responses are recently found to be important for innate and adaptive immunity particularly in parasitic infections. To date, the study on the role of IL-9 in bacterial infections is limited and the reported data are contradictory. One reported function of IL-9/Th9 is to modulate Th1/Th17 responses. Since our and others’ previous work has shown a critical role of Th1 and Th17 cells in host defense against chlamydial lung infection, we here examined the role of IL-9 responses in *Chlamydia muridarum* (Cm) lung infection, particularly its effect on Th1 and Th17 responses and outcome infection. Our data showed quick but transient IL-9 production in the lung following infection, peaking at day 3 and back to baseline around day 7. CD4+ T cell was the major source of IL-9 production in the lung infection. Blockade of endogenous IL-9 using neutralizing antibody failed to change Interferon-γ (IFN-γ) and IL-17 production by cultured spleen mononuclear cells isolated from Cm infected mice. Similarly, in vivo neutralization of IL-9 failed to show significant effect on T cell (Th1 and Th17) and antibody responses (IgA, IgG1 and IgG2a). Consistently, the neutralization of IL-9 had no significant effect on disease process, including body weight change, bacterial burden and histopathological score. The data suggest that IL-9 production following chlamydial lung infection is redundant for host defense against the intracellular bacteria.

## Introduction

IL-9 is a cytokine which has been identified and characterized for more than two decades[1]. IL-9 is involved in parasitic infections, allergy, and inflammatory processes[1]. Although initially considered as a Th2 cytokine, IL-9 can be produced by many other cells especially T cell subsets including Th1, Th17, Th22 and regulatory T (Treg). In particular, a specific CD4 T cell linage, Th9, is found to predominantly produce IL-9[2]. PU.1 and interferon regulatory factor 4 (IRF4) are the typical transcription factor for Th9 development. Differentiation of Th9 is dependent on the presence of IL-4 and transforming growth factor-β (TGF-β) in the microenvironment[3]. IL-25, a protein of IL-17 family, can also promote Th9 responses[2]. In addition to CD4 T cells, it is recently reported that innate lymphoid cells (ILC) could be a major source of IL-9 in certain lung inflammations[3]. IL-9 can affect various cell types, including T cells, mast cells, B lymphocytes and lung epithelial cells [4]. In particular, IL-9 is a pleiotropic cytokine influencing the differentiation of various T subset cells.

The influence of IL-9 on Th1 and Th17 cells has been frequently reported although some data are contradictory. IL-9 has effects on signal transducers and activators of transcription (STATs) which play critical roles in regulating helper/effector T-cell differentiation [5]. STAT4 can promote the production of signature Th1 cytokine, IFN-γ, which in turn activates STAT1 and induces expression of T-bet (the principal Th1 transcription factor) [6]. IL-9 can also induce tyrosine phosphorylation of STAT1, which is also important for the development of Th1 cells [7]. However, the promoting effect of IL-9 on Th1 cells appears infection/disease dependent. For example, Grohmann *et al*. showed that administration of exogenous IL-9 protected mice in a *Pseudomonas aeruginosa*-induced sepsis model [8]. Similarly, Finiasz *et al*. found that IL-9 could promote the production of IFN-γ and had a protective role in *Mycobacterium leprae* infection [9]. In contrast, Wu *et al*. found that IL-9 reduced IFN-γ mRNA expression in peripheral blood mononuclear cell (PBMC) and contributed to the development of *Mycobacterium tuberculosis* infections [10].

The promoting role of IL-9 on Th17 cells appears more consistent than Th1 response. Th17 differentiation depends on the activation of STAT3 [11]. IL-6 is a major trigger of STAT3 activation while a recent study has found that IL-9 treatment can induce tyrosine phosphorylation of STAT3 in T cells, thus promoting the differentiation of Th17 cells [12]. IL-6 can cooperate with TGF-β, playing a critical role of in the differentiation Th17 cells [13–15]. A recent study showed that IL-9 and TGF-β also could cooperatively promote the differentiation of Th17 cells in vitro [12]. Moreover, Th17 cells can secrete IL-9, which in turn stimulates the development of Th17 cells [16].

Chlamydiae are a group of obligate intracellular bacterial pathogen. Two chlamydial species, *Chlamydia trachomatis* and *Chlamydia pneumoniae*, can cause various human diseases. *C*. *pneumoniae* causes respiratory diseases like bronchitis, sinusitis and pneumonia, whereas *C*. *trachomatis* is a major cause of ocular and sexually transmitted diseases [17]. The mouse pneumonitis agent strain, recently designated as *Chlamydia muridarum* (Cm), has been widely used in mouse models of respiratory and genital tract infections [18]. Th1 response has long been demonstrated to be the dominant protective determinant for controlling chlamydial infection in human and mouse models [19–21]. More recently, our and others’ studies indicate that Th17 plays an important role in host defense against chlamydial infection, through either promoting Th1-type cell responses and/or working synergistically with Th1 cytokine, IFNγ [22]. Therefore, the development of both Th1 and Th17 cell immune responses is optimal for host defense against chlamydial lung infections. Considering the reported effect of IL-9 on Th1/Th17 cells, we examined, in the present study, IL-9 responses following chlamydial lung infection and the influence of this response on adaptive T cell and B cell responses as well as on the outcome of infection. Surprisingly, although significant IL-9 response was induced by Cm lung infection, the blockade of this cytokine in vivo failed to change either T cell or B cell responses and had no significant impact on infection process. The data suggest that IL-9 response is redundant for host defense against chlamydial lung infection.

## Materials and Methods

### Mice

Male C57BL/6 mice (6–8 weeks old) were purchased from the University of Manitoba animal care facility. The mice were hosted at a pathogen-free laminar flow cabinet. Mice were humanly sacrificed using 4% isoflurane if the weight drop more than 25% in our experiment. This study was carried out in accordance with the recommendations in the Canadian Council on Animal Care (CCAC) Guidelines and according to animal use protocols approved by the Protocol Management and Review Committee of University of Manitoba.

### Organism

*C*. *muridarum* organisms (Nigg strain) were cultured, purified and quantified as previously described [23]. In brief, *C*. *muridarum* was grown in HeLa 229 cells in RPMI 1640 medium supplemented with 10% fetal bovine serum (FBS), 1% L-glutamine and 25mg/ml gentamycin, and the elementary bodies (EBs) were purified by discontinuous density gradient centrifugation. The infectivity of purified EBs was measured by infecting Hela 229 and immunostaining of chlamydial inclusions. The purified EBs were suspended in sucrose-phosphate-glutamic acid (SPG) buffer and stored at -80°C. The same batch of purified EBs was used throughout this study.

### Mouse infection and treatment

Mice were intranasally inoculated with 1×10^3^ inclusion-forming units (IFU) of *C*. *muridarum* EBs in 40μl SPG buffer. For IL-9-blockade experiments, mice were intranasally administered with 10μg (40μl) of either Armenian hamster IgG anti-mouse IL-9 mAb (Cat:16–7093, eBioscience, San Diego, USA) or Armenian hamster IgG isotype control (Cat:16–4888, eBioscience, San Diego, USA) at one day before, the same day and every 2 days after the intranasal infection. Mice were sacrificed at designated days after infection. Lung tissues were removed and homogenized in 3ml SPG buffer. After centrifugation to clear residual debris, the homogenates were used for measuring cytokines (IL-9, IL-17 and IFN-γ) and titrating live chlamydial organisms as described [24].

### Analysis of lung pathology

The lung tissues of mice were perfused with PBS and fixed in 10% formalin. The tissue sections were routinely stained with H&E (hematoxylin and eosin) and examined under light microscopy as described[25]. The degree of lung inflammation was scored using a semi-quantitative grading system[26]: 0, normal; 1, mild and limited inflammation, granuloma formation and cellular infiltration in less than 25% of area, no obvious infiltration into adjacent alveolar septae or air space; 2, mild interstitial pneumonitis, diffused cellular infiltration in 25–50%) of area with septal congestion and interstitial edema; 3, inflammatory cell infiltration into perivascular, peribronchiolar, alveolar septae, and air space (50%–75% of area); 4, over 75% of area of lung filled with infiltrating cells.

### Lung, spleen and local lymph node (LN) cell isolation

Lung, spleen and draining LN cells were isolated for cytokine and Flow cytometry assay as described[27,28]. Briefly, lung tissues were harvested from the animals at specified time and digested in 2 mg/ml collagenase XI (Sigma-Aldrich, Oakville, Ontario, Canada) in RPMI 1640 for 1h at 37°C. After digestion, 35% (volume/volume) Percoll (Pharmacia, Uppsala, Sweden) and ACK lysis buffer (150mM NH_4_Cl, 10mM KHCO_3_, 0.1mM EDTA) were used to remove tissue debris and red blood cells (RBC), respectively. For spleen single-cell preparation, spleens were cut into small pieces and digested in 2mg/ml collagenase D (Roche Diagnostics, Meylan, France) in RPMI 1640 for 30 minutes at 37°C. The cell suspension was filtered and RBCs were removed by ACK lysis buffer. For LN mononuclear cell isolation, LNs were homogenized in 3ml RPMI 1640 and RBCs were removed by ACK lysis buffer. All of the cells were washed and resuspended in complete RPMI-1640 medium (RPMI-1640 supplemented with 10% FBS, 1% L-glutamine, 25mg/ml gentamicin and 0.05mM 2-mercaptoethanol). Single-cell suspensions were cultured in 48-well plates at a concentration of 7.5×10^6^ (spleen) and 5×10^6^ (lung and LN) cells/well with UV-inactivated Cm (1×10^5^ IFU/ml). The supernatants were collected from the cell cultures after 3 days and assayed for IFN-γ and IL-17 by enzyme-linked immunosorbent assay (ELISA) using antibodies purchased from eBioscience.

### Spleen cells cultured with anti-IL-9 mAb *in vitro*

Spleens were harvested from the mice at day 3 after Chlamydial lung infection. The spleen cells were digested and resuspended for further applications. Spleen cells were cultured at 7.5×10^6^ cells in 1 ml culture medium per well in 48-well plates with UV-inactivated-Cm stimulation in the presence of anti-mouse IL-9 mAb or Isotype control at different doses (0.5μg, 1.0μg, 2.0μg or 4.0μg) for 72 h. The supernatants were collected from the cell cultures and assayed for IFN-γ and IL-17 by ELISA using antibodies purchased from eBioscience.

### Flow cytometric analysis

The IFN-γ, IL-17 and IL-9 production by individual cells was analyzed by intracellular cytokine staining as described previously [25]. Briefly, the cells were stimulated with PMA (50 ng/ml) and Ionomycin (1μg/ml), and incubated at 37°C in complete RPMI 1640 medium. Two hours later, brefeldin A (eBioscience, San Diego, USA) was added to the culture, and the cells were cultured for another 4 h to accumulate cytokines intracellularly. The cells were collected and blocked with anti-CD16/CD32 Abs (eBioscience, San Diego, USA) in FACS buffer for 20 min and then surface stained with anti-CD3ε-PEcy7, anti-CD4-FITC, anti-CD45-FITC, anti-CD8α-PE, anti-NK1.1-PE and anti-CD1d tetramer-PE mAbs (eBioscience, San Diego, USA). After fixed and washed with permeabilization buffer, cells were stained with anti-IFNγ-allophycocyanin, anti-IL-17-allophycocyanin and anti-IL-9-allophycocyanin mAbs (eBioscience, San Diego, USA), or with corresponding isotype control Abs for 30 min. Cells were washed twice with permeabilization buffer and analyzed by flow cytometry. All of the data were collected using a LSR II flow cytometer (BD Biosciences, San Diego, USA) and analyzed using FACS express software (De Novo Software, Los Angeles, USA).

### Determination of Cm-specific Ab levels

Ab titers for Cm-specific IgA, IgG1 and IgG2a were measured using an alkaline phosphatase-based ELISA as previously described [21,29]. Briefly, microtiter plates were coated overnight with killed Cm elementary bodies (EBs). After blocking and washing, serially-diluted sera were incubated for 3 h at 37°C. Biotinylated goat anti-mouse Ab was added after washing. Following overnight incubation at 4°C, alkaline phosphatase-conjugated streptavidin (Jackson ImmunoResearch Laboratories; Bio/Can Scientific) was added for 45 min at 37°C. The enzyme substrate *p*-nitrophenyl phosphate (Sigma-Aldrich) (in 0.5 mM MgCl_2_, 10% diethanolamine (pH 9.8)) was added and the reaction was allowed to proceed for 60 min. The plates were read by using an ELISA reader at 405 nm. Results were expressed as ELISA titers using the endpoint (cutoff at OD 0.5) of the titration curves.

### Statistical analysis

Statistical analysis of the data was performed using ANOVA and *t* tests (GraphPad Prism software, version 5.0), values of p<0.05 were considered significant. Data are presented as mean±SD. The presented data were collected from all the 4 mice of each group. All the experiments were repeated two to three times with similar results.

## Results

### Cm lung infection induces IL-9 production

**Fig 1:**
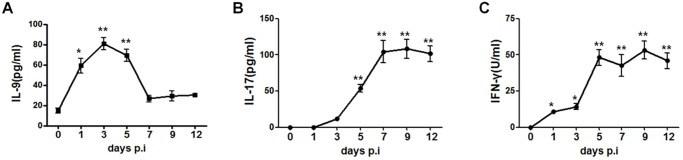
Kinetics of local IL-9, IL-17 and IFN-γ production following lung Cm infection. C57BL/6 Mice were inoculated intranasally with Cm (1×10^3^ IFUs) and were sacrificed at the indicated days. The lungs were isolated and the lung homogenates was prepared to detect IL-9(A), IL-17(B) and IFNγ (C) protein levels by ELISA. One representative experiment of two independent experiments (4 mice in each group) is shown. The results are shown as mean ± SD. The levels of IL-9, IL-17 and IFN-γ at various days post-infection were compared to those on day 0 by ANOVA (*p<0.05;**p<0.01).

**Fig 2:**
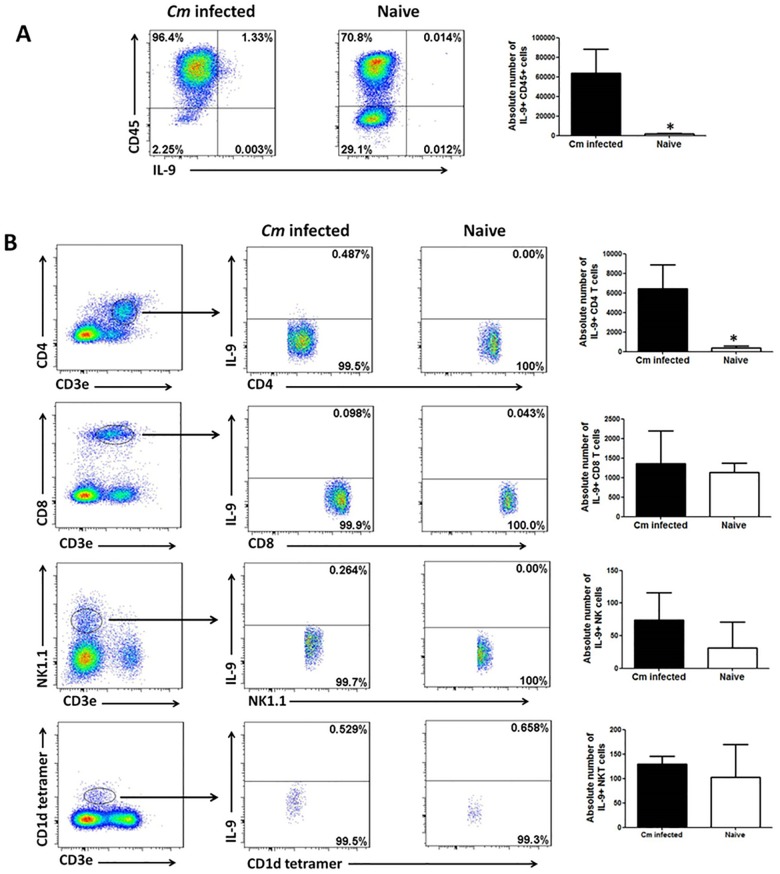
Production of IL-9 by lung mononuclear cells following Cm infection. Mice were inoculated intranasally with Cm (1×10^3^ IFUs) and were sacrificed at day 3 post-infection. The isolated lung mononuclear cells were detected for IL-9 expression by FACS. (A) Representative intracellular IL-9 staining of CD45+ cells and absolute number of IL9 producing CD45+ cells in the lung. (B) Representative intracellular IL-9 staining and absolute number of IL-9 producing CD4+ (CD4+CD3+) T cells, CD8+ (CD8+CD3+) T cells, NK (NK1.1+CD3-) cells and NKT (CD1d tetramer+NK1.1+) cells in the lung. One representative experiment of two independent experiments (four mice in each group in each experiment) is shown. The results are shown as mean ± SD (*, p＜0.05).

We first examined IL-9 response following Cm lung infection. The data showed quick increase of IL-9 levels in the lung of the infected C57BL/6 mice, almost reaching the peak on day 1 (Fig. 1A). The IL-9 levels reached the maximum level on day 3. On day 5, the level of IL-9 started to drop and virtually got back to baseline on day 7. The lung expression of IL-17 and IFN-γ messages were also measured at the same time points. As shown in Fig. 1B&C, the expression of IL-17 and IFN-γ in the lung increased much slower than IL-9 following intranasal Cm infection, becoming detectable at day 1 and peaking at day 7–12. The results suggest that IL-9 can potentially influence IL-17 and IFN-γ responses in Cm infection. To further identify the source of IL-9 production, we performed intracellular cytokine staining on various lung lymphocytes. We used markers for innate and adaptive immune cells potentially producing IL-9. We found significant increase of IL-9 producing CD45+ lymphocytes following infection at day 3 post-infection, in both percentages and absolute cell numbers (Fig. 2A). Further analysis of the various types of lymphocytes showed that CD4+ T cells (CD4+CD3ε+) was the major producer of IL-9 (Fig. 2B). The other tested lymphocytes, CD8+ T cells (CD8+ CD3ε+), NK cells (NK1.1+ CD3ε-) and NKT cells (CD1d tetramer+ CD3ε+) were not significant IL-9 producers (Fig. 2B). The kinetics and intracellular cytokine data show a significant and quick, but transient, local production of IL-9 by CD4+ T cells following Cm lung infection.

### Blockade of IL-9 failed to alter IFN-γ and IL-17 production in the culture of ex vivo spleen cells from infected mice

**Fig 3:**
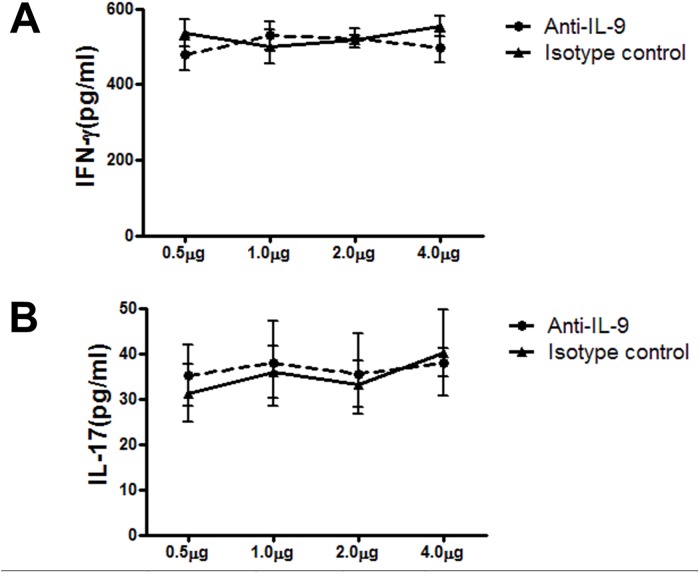
Blockade of endogenous IL-9 had no significant effect on IFN-γ and IL-17 production by ex vivo spleens isolated from Cm infected mice. Spleen cells from mice infected with Cm at day 3 post-infection were cultured in 48-well plates with UV-inactivated-Cm stimulation in the presence of anti-mouse IL-9 mAb or isotype control at various concentrations as indicated. The culture was proceeded for 72 hours and the concentration of IFN-γ and IL-17 in culture supernatants was determined by ELISA. One representative experiment of three independent experiments is shown. The results are shown as mean± SD of each group.

To directly assess the role of IL-9 in the production of IFN-γ and IL-17 following Cm infection, we first used neutralizing anti-mouse IL-9 mAb to block IL-9 in culture of ex vivo spleen cells, which were isolated from Cm infected mice at day 3. The cells was stimulated with UV-inactivated Cm in the presence of different concentrations of anti-IL-9 mAb or isotype control; and the IFN-γ and IL-17 levels of in the culture supernatants at 72 h were determined by ELISA. The results showed that the added IL-9 mAb did not affect the production of IFN-γ and IL-17 by the cultured cells (Fig. 3).

### The *in vivo* neutralization of IL-9 had no effect on Th1 response following Cm infection

**Fig 4:**
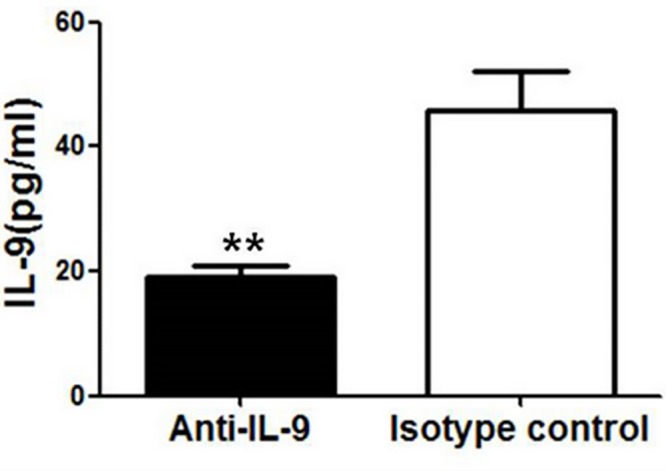
Intranasal delivery of anti-IL-9 mAb successfully neutralized IL-9 in the lung. C57BL/6 mice were intranasally administered with 10μg of either anti-mouse IL-9 mAb or isotype control antibody at day -1 (one day before), day 0, day 2 following Cm (1×10^3^ IFUs) lung infection. On day 3 post-infection, mice were sacrificed and lung homogenates were prepared to detect IL-9 protein by ELISA. One representative experiment of two independent experiments (four mice in each group in each experiment) is shown. The results are shown as mean ± SD (**, p＜0.01).

**Fig 5:**
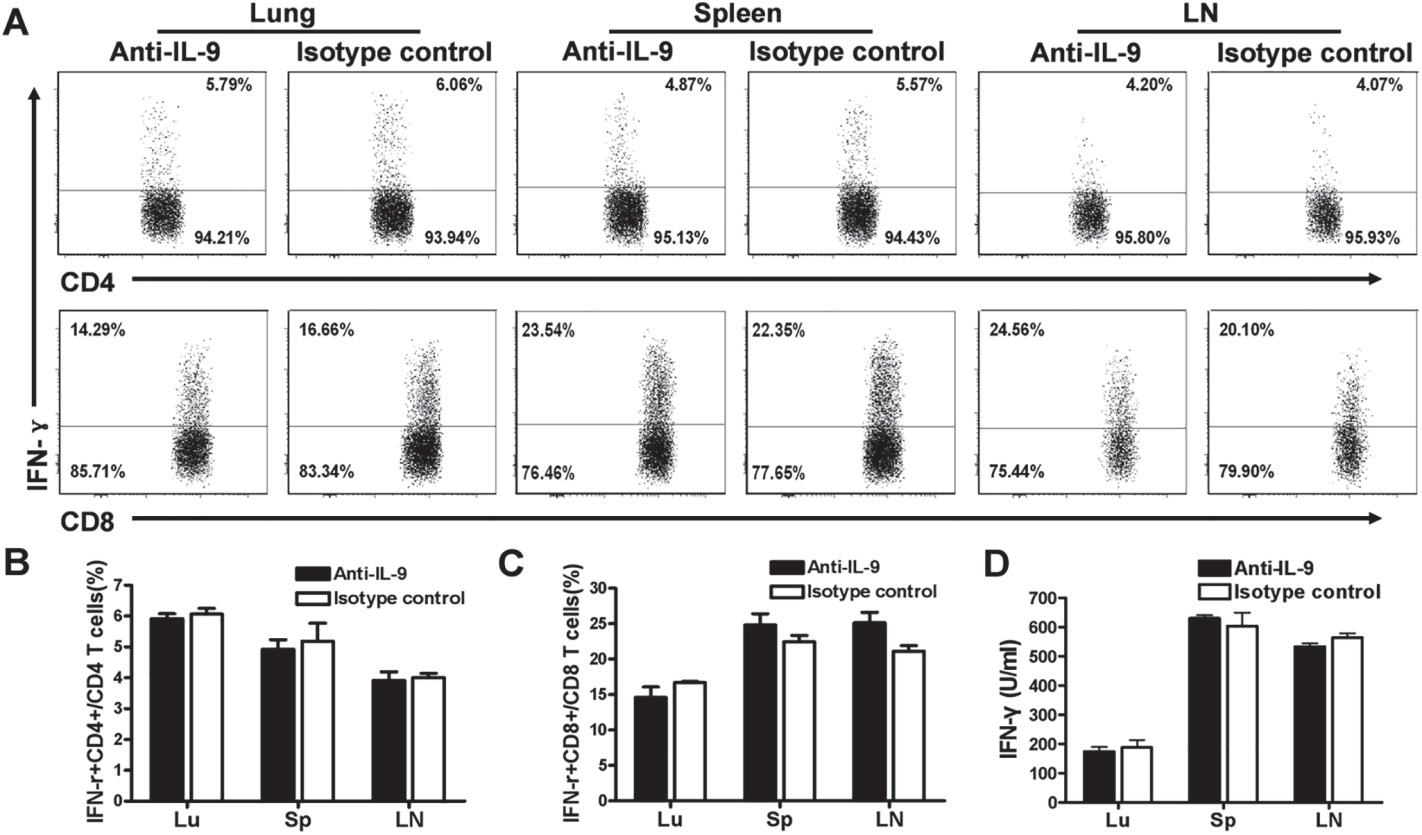
In vivo neutralization of IL-9 failed to alter IFNγ producing by CD4+ and CD8+ T cells following Cm lung infection. C57BL/6 mice (4 mice per group) were treated with anti-IL-9 mAb or isotype control antibody at days -1, 0, 2, 4 and 6 following intranasal infection with Cm (1×10^3^ IFUs) and sacrificed at day 7 after infection. (A) Representative data of IFN-γ-producing CD4+ and CD8+ T cells in each group. (B) & (C) Summary of the percentage of IFN-γ+ CD4+ T cells and IFN-γ+ CD8+ T cells in each group (4 mice/group in each experiment). (D) Lung, spleen and draining LN cells were isolated and cultured with UV-inactivated EBs. The concentrations of IFN-γ in the culture supernatants were measured by ELISA. One representative experiment of three independent experiments (4 mice in each group) with similar results is shown. Data are shown as the mean ± SD.

To further assess the potential role of IL-9 in the development of Th1 response in vivo, we used the anti-IL-9 mAb to treat mice before and after Cm lung infection. We first confirmed if the antibody treatment could neutralize IL-9 in vivo. As shown in Fig. 4, the treatment with anti-IL-9 mAb, but not isotype control, effectively knocked down IL-9 levels by ~60%. We then compared IFNγ production by CD4+ and CD8+ T cells isolated from the lung, spleen and draining LNs of anti-IL-9-treated and isotype control antibody treated mice at day 7 post-infection. As shown in Fig. 5 (A, B and C), no significant difference was found by intracellular staining on the frequencies of IFNγ-producing CD4+ or CD8+ T cells between IL-9-neutralized mice and isotype control mice in the different organs. To further confirm this in population level, we did ELISA analyses of IFN-γ production by the cultured lung, spleen and LN mononuclear cells isolated from the different groups (Fig. 5D). Consistently, no significant difference was found between the groups in the different organs. Of note, similar results were found on the analysis at day 14 post-infection (data not shown). These results suggest that IL-9 production induced by Cm infection had no significant effect on Th1 response during chlamydial lung infection.

### The neutralization of IL-9 did not affect Th17 immune response in Cm lung infection

**Fig 6:**
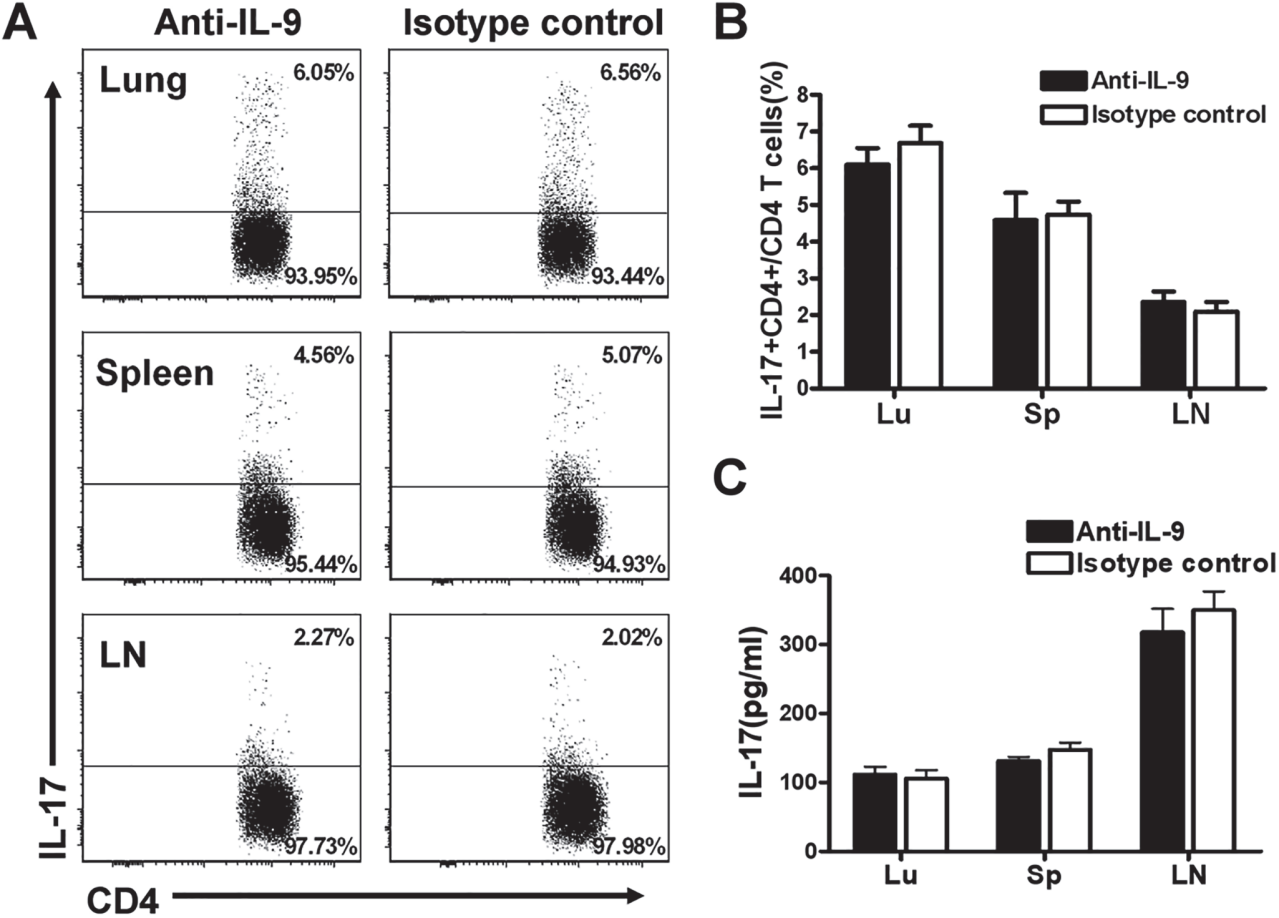
In vivo neutralization of IL-9 failed to alter IL-17/Th17 production following Cm lung infection. The mice were treated as described in legend to Fig. 5. On day 7 post-infection, lung, spleen and draining LN cells were isolated and analyzed by intracellular cytokine staining or cultured for 72 h following by ELISA testing of IL-17. (A) Representative staining of IL-17 producing CD4 cell in the lung, spleen and draining LNs; the percentages of IL-17-producing CD4+ T cells were showed in the right upper quadrant of the graph. (B)Summary of the percentage of IL-17 producing CD4 T cells in each group. (C) Lung, spleen and draining LN cells were cultured with stimulation of UV-inactivated Cm EBs, and IL-17 concentrations in 72h culture supernatants were determined by ELISA. Three independent experiments with four mice in each group were performed, and one representative experiment is shown. Data show the mean ± SD.

Our previous work found that IL-17 played a protective role in host defense against chlamydial infection and more importantly worked synergistically with Th1[22]. We therefore also examined IL-17/Th17 responses in the IL-9 blocked mice. Intracellular cytokine-staining analyses showed that there was no significant difference in IL-17-producing CD4+ T cells the in lung, spleen and LN between IL-9-neutralized mice and isotype control mice at day 7 post-infection (Fig. 6, A and B). The same is true on day 14 p.i (data not shown). At the population level, ELISA testing also showed similar levels of IL-17 production between IL-9 neutralized mice and isotype control treated mice by the cells isolated from lung, pleen and draining LNs (Fig. 6C). These data indicated that IL-9 production had no significant effect on IL-17/Th17 responses following Cm lung infection.

### IL-9 production has no significant effect on Cm-specific Ab responses

**Fig 7:**
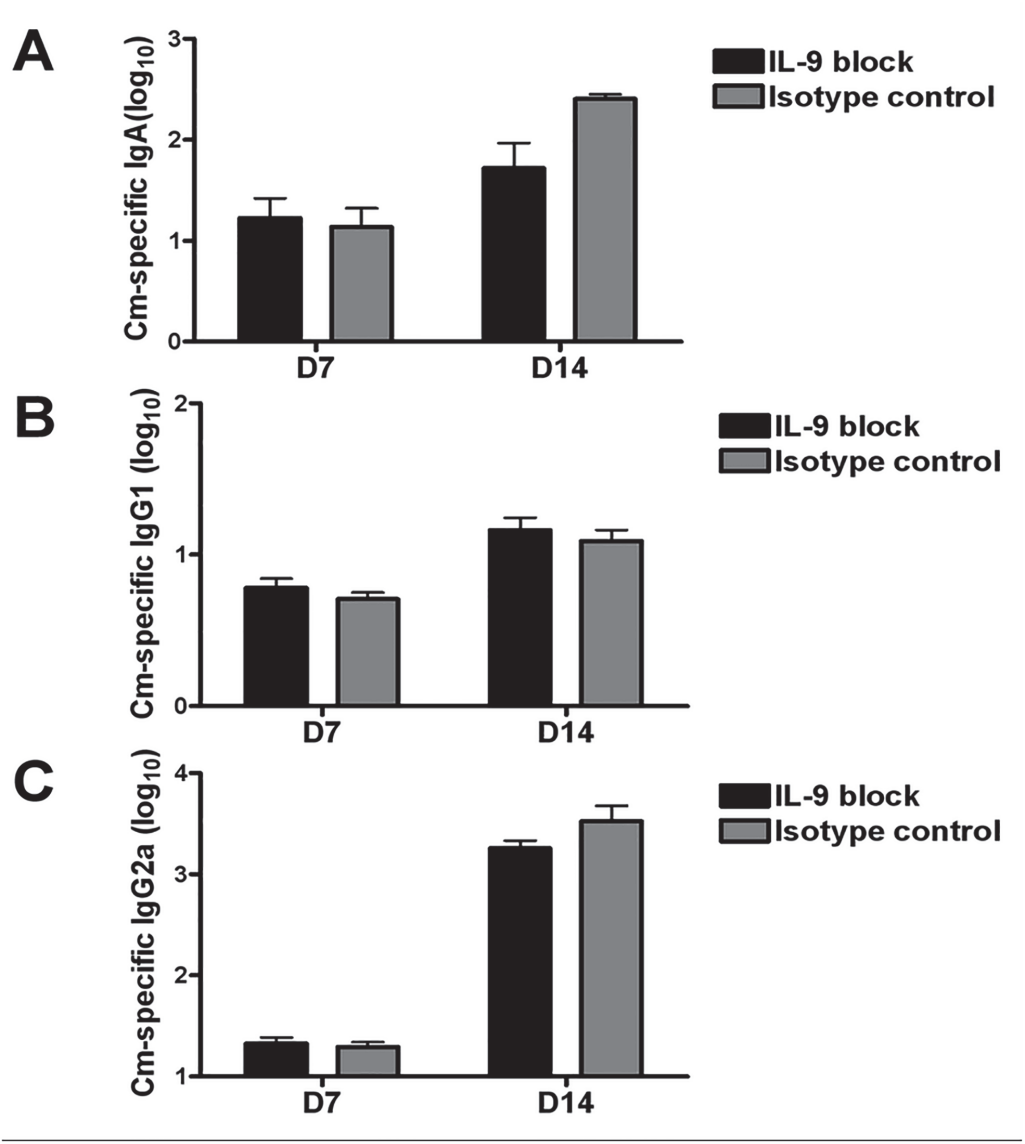
In vivo neutralization of IL-9 failed to alter serum Ab responses following Cm lung infection. Mice were treated 10 μg anti-IL-9 mAb or isotype control at days -1, 0 and every two day thereafter following intranasal Cm (1×10^3^ IFUs) infection. Serum samples were collected at days 7 and 14 post-infection and tested for *Cm*-specific IgA (A), IgG1 (B) and IgG2a (C). Ab titers were transformed to log 10 values. Pooled data for three experiments (12 mice in each group) are presented. Data is shown as mean ± SD.

We further measured the levels of Cm-specific IgA, IgG1 and IgG2a Abs in the sera collected from IL-9-neutralized and isotype control mice after infection. Following lung infection (day 7 and day 14), the IL-9-neutralized mice showed the similar levels of serum Abs (IgA, IgG1 and IgG2a) to the isotype control mice (Fig. 7). These results suggest that IL-9 had no significant influence on the production of the different isotypes of Cm-specific Abs following chlamydial lung infection.

### IL-9 did not contribute to protection against Cm lung infection

**Fig 8:**
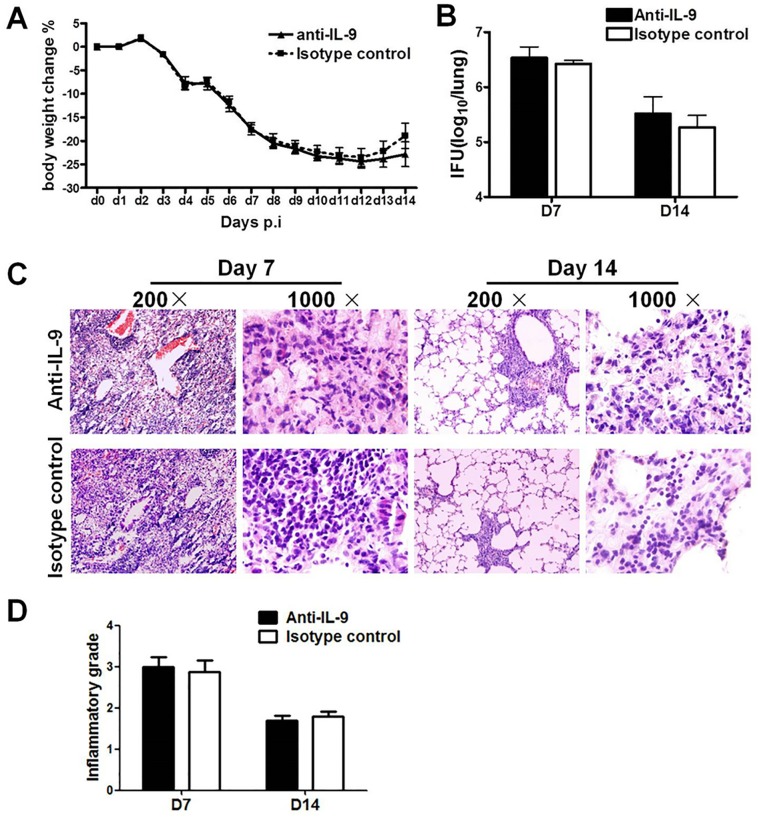
The neutralization of endogenous IL-9 has significant impact on infection process and histopathology in the lung. C57BL/6 mice were intranasally administered with 10μg of either anti-mouse IL-9 mAb or isotype control antibody at one day before and the same day of infection following by every 2 days thereafter. Mice were inoculated intranasally with 1×10^3^ IFUs of Cm EBs. (A) The percentage of body weight changes in the two groups of mice. (B) Live chlamydial organisms (IFUs) in the lung on day 7 and day 14 after infection in each group. (C) The lung tissue sections from different experiment groups were tained with H&E and observed under light microscopy. (D) Semi-quantitative analysis of lung inflammation and damage (pathological score). Slides were examined by a blinded pathologist and the inflammatory grades were analyzed as described in Materials and Methods. One representative experiment of three independent experiments (four mice in each group in each experiment) is shown. The results are shown as mean ± SD.

To examine whether IL-9 contributes to host defense against Cm infection in the lung, we tested the effect of anti-IL-9 mAb treatment on mouse body weight, bacterial burden and histopathology. Consistent with the findings on T cell and B cell responses, the neutralization of IL-9 did not affect these disease parameters in the infected mice. Specifically, no significant difference was found in bodyweight loss of IL-9-neutralized group compared to isotype control group (Fig. 8A). The bacterial loads (inclusion forming units, IFUs) in the lung were similar between the groups at both early (day 7) and later (day 14) stages of infection (Fig. 8B). Moreover, the results of histological analyses of the lung showed similar pattern and degree of histological inflammations and tissue damage in IL-9-neutralized and isotype control groups (Fig. 8C&D). The results suggest that the production of IL-9 in chlamydial lung infection is redundant for host defense, at least in this primary infection model.

## Discussion

In this work, we analyzed the kinetics of IL-9 production in the lung tissues following intranasal infection with Cm and tested the influence of the endogenous IL-9 production on T cell and B cell responses as well as disease outcomes using in vitro and in vivo antibody neutralization approaches. We found lung Cm infection did induced IL-9 production by CD45+ and more specifically CD4+ T cells (CD4+CD3ε+). The response is quick and transient in nature, almost reaching to peak at 24 h post-infection. The response maximized on day 3 with a quick decline within first week of infection. Interestingly, either in vitro or in vivo neutralization of the IL-9 activity failed to alter the Th1 and Th17 responses elicited by Cm lung infection. More importantly, the neutralization of IL-9 in vivo did not show significant impact on the process of primary lung infection and pathological changes. The results suggest that IL-9 production is redundant for host defense against chlamydial lung infection, at least in the primary infection model.

The kinetics of IL-9 response observed in this study is very different from most of the other cytokines which has been reported in the mouse lung infection model. Most cytokine responses reach maximum levels at the peak infection, 7–10 days post infection and some maintain in high level for even longer period of time. In contrast, IL-9 reaches to peak within 3 days and drop quickly when the infection is still increasing. The finding suggests that the IL-9 producing CD4+ T cells in this model are likely a type of innate cells which respond quickly in Cm lung infection. However, they were unlikely NKT cells because the specific analysis of iNKT cells did not show significant increase of IL-9 producing iNKT cells after infection although a certain level of baseline IL-9 production by iNKT cells exists (Fig. 2). Since the peak of IL-9 production is much earlier than IL-17 and IL-4 production by CD4 T cells, the IL-9 producing cells are not Th2 or Th17 cells either. Therefore, they are more likely Th9 cells although the current data is not sufficient to make this conclusion because there was no multiple cytokine analysis in a single cell level. Notably, the kinetics of IL-9 response in this infection model fits that observed in a hen egg lysozyme (HEL) immunization model, showing peaks of IL-9 at day 3 and subsequent sharp decline [30].

The very transient nature of IL-9 production could be one of the reasons for the insignificance of this cytokine in infection. Based on literature review of other models and infections, we initially hypothesized that IL-9 plays either a protective or detrimental role in host defense against chlamydial lung infection. On the one hand, IL-9 can induce the activation of STAT1 transcriptional factor, which is important for the development of Th1 cells and the production of IFN-γ [7]. Moreover, in the presence of TGF-β, IL-9 can differentiate naïve CD4+ T cells into Th17 cells in vitro, independent of IL-6 signaling[12]. Furthermore, the Th17 cell has the ability to produce IL-9 both in vitro and in vivo [31], in turn amplifying Th17 cells [32]. Further, Elyaman *et al*. indicated that addition IL-9 can promote IL-17 production by purified CD4 T cells in vitro[12]. Since Th1 and Th17 responses are critical for protection against Cm lung infection, the potential promoting effect of IL-9 on Th1 and Th17 may enhance protection. On the other hand, a regulatory role of IL-9 on IFNγ production has also been reported in some intracellular and extracellular infections, including *Mycobacterium tuberculosis*[10], *Pseudomonas aeruginosa*[8] and *Mycobacterium leprae* [9]. However, the data from our present study did not support either role of IL-9 in chlamydial infection. It further emphasizes the complexity of the role of cytokine and T cell subsets in infection and the importance to study specific infection models. The quick decline of IL-9 responses may be related to a feedback mechanism which quickly inhibits IL-9 production thus being used as escape strategy taken by chlamydia. In line with this though, we have found significant IL-10 and Treg responses shortly after the peak of IL-9 response (Peng et al, unpublished data), which may be involved in the quick reduction of IL-9. Further understanding on the reason of quick decline of IL-9 and intervention to maintain or enhance IL-9 may provide a new way to enhance protective immunity or reduce immunopathology in chlamydial infection.

The lack of effect of anti-IL-9 treatment on Th1/Th17 responses is obviously not because of a possible failure to neutralized IL-9 in the experiments. We have tested the role of IL-9 by both in vitro and in vivo approaches. In the in vitro study (Fig. 3), we used different concentrations of antibody upto 4 μg/ml but did not see any trend of changes in IFNγ and IL-17 production. More importantly, in the ex vivo experiments, we found that the protocol used for administration of anti-IL-9 mAb significantly neutralized IL-9 in the lung (Fig. 4) but failed to have any impact on T cell and B cell responses and protection. Notably, Elyaman et al’s study (12) examined the role of IL-9 on Th1/Th17 responses in a culture system of purified CD4 T cells with polyclonal stimulation. They found that the addition of recombinant of IL-9 had clear positive impact on Th1/Th17 responses in the particular system. However, we found that in chlamydial infection, either infecting ex vivo spleen cells or intact mice intranasally, IL-9 had no significant effect on Th1/Th17 responses. Since Cm mainly infect monocytes/macrophages and epithelial cells and activate T cells through antigen presenting cells, the potential role of IL-9 on T cells may be substituted by other factors produced by different cells which are infected by or influenced by Cm. This could be one of the reasons for the difference observed between our study and others’. The results further emphasize the importance to examine the role of particular cytokines in host defense against specific infections and the relevance of research models for addressing scientific questions.

Although the study failed to find significant impact of IL-9 on humoral and cellular immune responses as well as the outcome of primary chlamydial lung infection, it does not necessarily mean that IL-9/Th9 responses is completely useless. It still can play a role in some scenarios of chlamydial infection. For example, in a condition of immune deficiency of certain molecules or cells, IL-9 may become a replacement thus playing a compensatory role in host defense mechanisms. It may also play a role in the individuals with different genetic background or in the infections in other locations such as genital tract. More importantly, IL-9 may play a role in secondary infection involving the development and maintenance of immune memory. Therefore, further study is warranted to test the different possibilities.

Collectively, our study provides new insights into IL-9 response in the lung infection of *Chlamydiae*, an obligate intracellular bacteria. We have demonstrated quick and transient IL-9 production in the area of infection but failed to find a significant role of the endogenous IL-9 production on T cell and B cell responses related to host defense against chlamydial infection. The results suggest that the production of IL-9 in primary chlamydial lung infection is redundant for the development of adaptive immunity and protection.

## Supporting Information

S1 ARRIVE Checklist (PDF)
